# Hypermutation-induced *in vivo* oxidative stress resistance enhances *Vibrio cholerae* host adaptation

**DOI:** 10.1371/journal.ppat.1007413

**Published:** 2018-10-30

**Authors:** Hui Wang, Xiaolin Xing, Jipeng Wang, Bo Pang, Ming Liu, Jessie Larios-Valencia, Tao Liu, Ge Liu, Saijun Xie, Guijuan Hao, Zhi Liu, Biao Kan, Jun Zhu

**Affiliations:** 1 Department of Microbiology, Nanjing Agricultural University, Nanjing, China; 2 State Key Laboratory for Infectious Disease Prevention and Control, National Institute for Communicable Disease Control and Prevention, Chinese Center for Disease Control and Prevention, Beijing, China; 3 Department of Microbiology, Perelman School of Medicine, University of Pennsylvania, Philadelphia, United States of America; 4 Department of Biotechnology, Huazhong University of Science and Technology, Wuhan, China; Northwestern University, Feinberg School of Medicine, UNITED STATES

## Abstract

Bacterial pathogens are highly adaptable organisms, a quality that enables them to overcome changing hostile environments. For example, *Vibrio cholerae*, the causative agent of cholera, is able to colonize host small intestines and combat host-produced reactive oxygen species (ROS) during infection. To dissect the molecular mechanisms utilized by *V*. *cholerae* to overcome ROS *in vivo*, we performed a whole-genome transposon sequencing analysis (Tn-seq) by comparing gene requirements for colonization using adult mice with and without the treatment of the antioxidant, N-acetyl cysteine. We found that mutants of the methyl-directed mismatch repair (MMR) system, such as MutS, displayed significant colonization advantages in untreated, ROS-rich mice, but not in NAC-treated mice. Further analyses suggest that the accumulation of both catalase-overproducing mutants and rugose colony variants in NAC^-^ mice was the leading cause of *mutS* mutant enrichment caused by oxidative stress during infection. We also found that rugose variants could revert back to smooth colonies upon aerobic, *in vitro* culture. Additionally, the mutation rate of wildtype colonized in NAC^-^ mice was significantly higher than that in NAC^+^ mice. Taken together, these findings support a paradigm in which *V*. *cholerae* employs a temporal adaptive strategy to battle ROS during infection, resulting in enriched phenotypes. Moreover, *ΔmutS* passage and complementation can be used to model hypermuation in diverse pathogens to identify novel stress resistance mechanisms.

## Author summary

Cholera is a devastating diarrheal disease that is still endemic to many developing nations, with the worst outbreak in history having occurred recently in Yemen. *Vibrio cholerae*, the causative agent of cholera, transitions from aquatic reservoirs to the human gastrointestinal tract, where it expresses virulence factors to facilitate colonization of the small intestines and to combat host innate immune effectors, such as reactive oxygen species (ROS). We applied a genome-wide transposon screen (Tn-seq) and identified that deletion of *mutS*, which is part of DNA mismatch repair system, drastically increased colonization in ROS-rich mice. The deletion of *mutS* led to the accumulation of catalase-overproducing mutants and a high frequency rugose phenotype when exposed to ROS selective pressures *in vivo*. Additionally, ROS elevated mutation frequency in wildtype, both *in vitro* and *in vivo*. Our data imply that *V*. *cholerae* may modulate mutation frequency as a temporal adaptive strategy to overcome oxidative stress and to enhance infectivity.

## Introduction

*Vibrio cholerae*, the etiological agent of the pandemic disease cholera, resides in aquatic environments and can also colonize human intestines following ingestion of contaminated food and water. In order to survive in both aquatic and host environments, *V*. *cholerae* has the ability to cope with harsh conditions during the transition to the host gut and during subsequent growth [1]. For example, upon infection, *V*. *cholerae* senses host signals and is able to coordinate both virulence gene activation and repression to evade host defenses and successfully colonize intestines [2–5]. Late in the infection, *V*. *cholerae* also optimally modulates its genetic programs for the forthcoming dissemination into the aquatic environment [6, 7] where it is often associated with abiotic or biotic surfaces such as phytoplankton and zooplankton. These associations enable the formation of biofilms, which provide protection from a number of environmental stresses; including nutrient limitation, protozoa predation, and bacteriophage infection [8]. Additionally, biofilms may enhance infectivity due to their acid-resistant properties and higher growth rate during infection [9, 10].

One of the major stresses *V*. *cholerae* must overcome is exposure to reactive radical species. Reactive compounds, including reactive oxygen species (ROS), are abundant in marine systems [11]. *V*. *cholerae* also encounters oxidative stress during the later stages of infection, as demonstrated by an increase in ROS levels and a decrease in the levels of host antioxidant enzymes during *V*. *cholerae*-induced diarrhea [12, 13]. It has been previously demonstrated that catalases (KatG and KatB), peroxiredoxin (PrxA), organic hydroperoxide resistance protein (OhrA), a redox-regulated chaperone (Hsp33), and a DNA-binding protein from starved cells (DPS) are important for *V*. *cholerae* ROS resistance [14–17]. ROS resistance in *V*. *cholerae* is known to be tightly regulated through a variety of mechanisms. OxyR is required to activate catalase genes and *dps*, and is modulated by another OxyR homolog, OxyR2 [14, 16, 18]. Quorum sensing systems [19], PhoB/PhoR two-component systems [20], and the virulence regulator, AphB, also play important roles in oxidative stress response [21]. Further identifying bacterial stress responses to host-derived ROS is important for understanding *V*. *cholerae* pathogenesis.

In this study, we used Tn-seq to screen for *V*. *cholerae* genes that are involved in ROS resistance during infection. By comparing colonization in control mice to mice treated with antioxidant N-acetyl cysteine (NAC) that reduces the production of ROS in murine intestines [15], we found that deletion of *mutS*, encoding a key component in the DNA methyl-directed mismatch repair (MMR) system, results in a significant colonization advantage compared to wildtype in ROS-rich mice. The MMR system is highly conserved from bacteria to humans and is critical for maintaining the overall stability of the genetic material [22]. Mutations in this pathway lead to hypermutation rates across the genome. It has been shown that inactivation of the MMR system of various bacterial pathogens, such as *Escherichia coli*, *Salmonella enterica* serovar Typhimurium, and *Pseudomonas aeruginosa* leads to better adaptation and persistence of these pathogens in murine models [23–26]. It has been proposed that under certain stressful conditions, hypermutators are selected in the total population by hitchhiking with the adaptive mutations that they produce. However, the mechanism(s) by which hypermutators become better persistors is less clear. In this work, we developed a strategy to study bacterial temporal hypermutation *in vivo* and found that mutations resulting in increased catalase production and increased biofilm formation, demonstrated by rugose colony phenotypes, may lead *V*. *cholerae* hypermutators to display colonization advantages in ROS-rich mouse intestines.

## Results

### Tn-seq screens identify *in vivo* enrichment of mutations in MMR pathways in the presence of ROS

**Fig 1:**
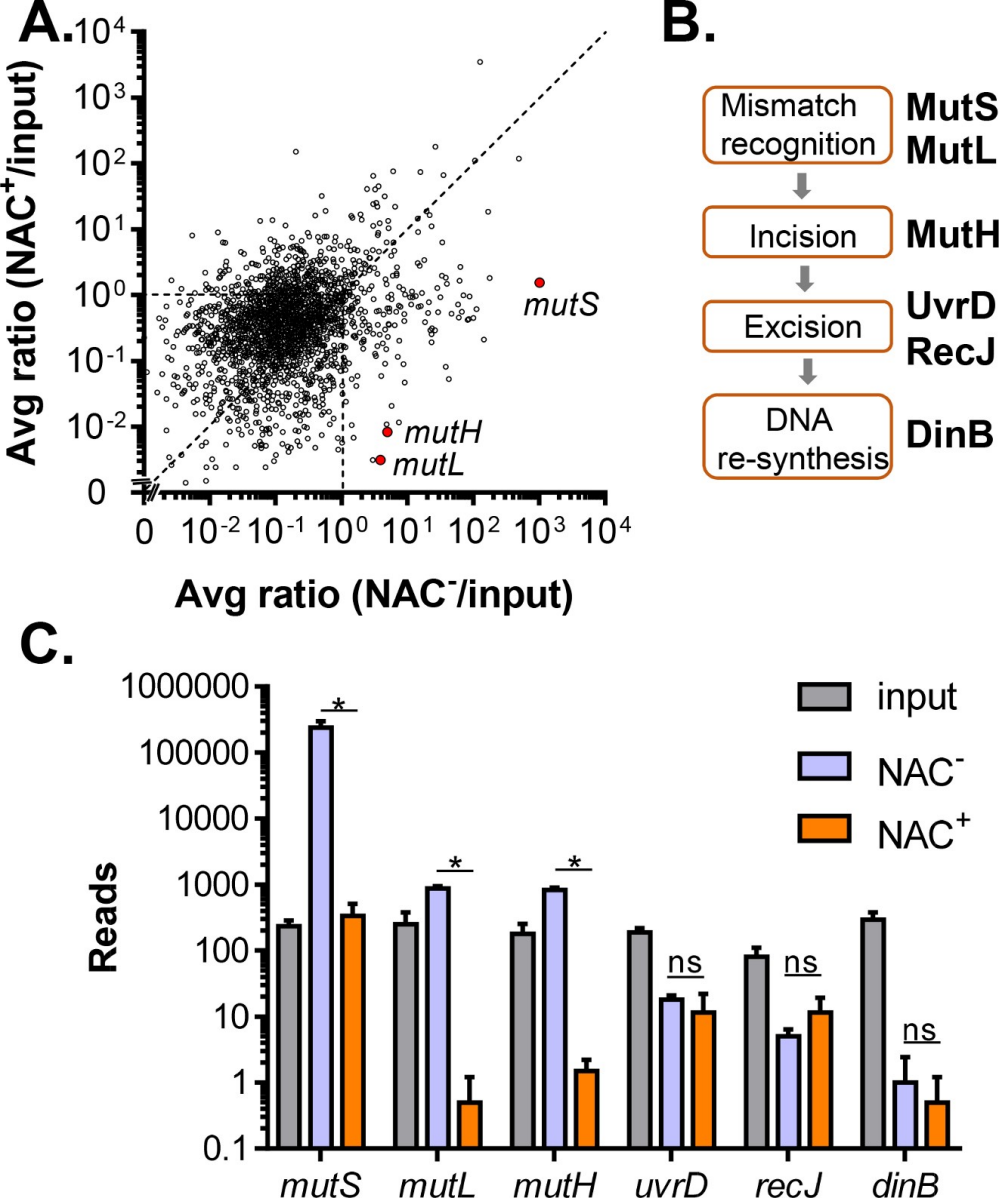
Tn-seq identification of the enrichment of DNA mismatch repair pathway mutants in NAC^-^ mice. **A.** Tn-seq results. Average of output/input ratios from two Tn libraries of mapped read counts of Tn mutants pooled from five mice without N-acetyl cysteine (NAC) treatment (NAC^-^ mice) were normalized against average of output/input ratios from two Tn libraries of those from NAC-treated mice (NAC^+^ mice)(pooled from five mice each group). **B.** DNA mismatch repair system pathway. **C.** Selected average mapped read counts of Tn mutants in the DNA mismatch repair pathway. Error bars represent means and SDs from two independent libraries. *: Student t-test, P < 0.05. ns: no significance.

To investigate *V*. *cholerae* genes involved in ROS resistance during colonization, we performed a Tn-seq screen in a streptomycin-treated adult mouse model, in which bacteria experience host-generated oxidative and nitrosative stress [15, 27, 28]. As a comparison, we also treated a set of mice with N-acetyl cysteine (NAC), an antioxidant widely used in human and animal studies to artificially reduce ROS levels [29, 30]. Previously we have shown that NAC significantly reduces the production of ROS related biomarkers in mice [15]. We mutagenized *V*. *cholerae* with a Tn5 transposon and inoculated the Tn5 library into adult mice with NAC treatment as a variable. At the 3-day post-infection (PI) time point, passaged mutants were recovered from fecal pellets. We then extracted bacterial DNA and used Illumina sequencing [6] to determine the number of transposon insertions in the input and output mutant libraries. We compared the output/input ratios of mutants colonized in NAC-treated mice (NAC^+^ mice) to mice without NAC treatment (NAC^-^ mice) (Fig 1A). Several mutations that have Tn insertions in previously-known genes required for ROS resistance were found colonizing poorly in NAC^-^ mice but not in NAC^+^ mice (S1 Data), validating the NAC treatment and suggesting that these genes are important for overcoming ROS *in vivo*. These genes include *prxA* (VC2637)[14], *ohrA* (VCA1006)[15], *dps* (VC0139)[16], and *rpoS* (VC0534)[21]. In addition, we identified iron transport systems (VC0776-VC0780, VC1264), efflux pumps (VC0629, VC1410, VC1675, VC2761, VCA0183, VCA0267), and a number of transcriptional regulators (such as VC0068, VC2301, VCA0182) that are important for colonizing in NAC^-^ mice (S1 Data). These genes are subject for independent confirmation and further investigation.

Interestingly, the Tn-seq screen revealed that a number of mutations are highly enriched in NAC^-^mice but not in NAC^+^ mice (Fig 1A), suggesting that mutants containing disruptions in these genes have colonization advantages in ROS-rich intestines. Among them, several mutations in DNA methyl-directed mismatch repair (MMR) pathways displayed significantly higher number of reads in the pools isolated from NAC^-^ mice than those of NAC^+^ mice (Fig 1A). MMR is highly conserved in all organisms and repairs mispaired bases in DNA generated by replication errors [22]. In *E*. *coli*, MutS recognizes mispairs and coordinates with MutL and MutH to direct excision of the newly synthesized DNA strand [31](Fig 1B). We found that the reads of insertions in *mutS*, *mutL*, and *mutH* from NAC^-^ mice were all higher when compared to NAC^+^ mice, whereas reads of insertions in the downstream MMR pathway (*uvrD*, *recJ* and *dinB*) were similar between these two conditions (Fig 1C). It has been reported that UvrD, RecJ, and DinB play less critical roles in bacterial DNA repair than MutSLH [22, 32]. We confirmed that in *V*. *cholerae*, deletion of *dinB* did not affect colonization, nor spontaneous mutation frequency (Fig A in S1 Text). Therefore, in this study, we selected MutS for further investigation to decipher the possible role of hypermutation on ROS resistance. Of note, the Tn-seq screen also revealed that other mutations are significantly enriched in NAC^-^ but not in NAC^+^ mice. These mutations included genes in the flagellar biosynthesis pathway (VC2120-VC2134) and the MSHA pilin biogenesis pathway (VC0398-VC0411)(S1 Data). The mechanisms are subjected to another study, but we speculate that since both flagella and MSHA pilins activate host innate immunity [2, 33, 34], which is activated by reactive oxygen species synergistically [35, 36], deletion in flagellar synthesis or MSHA synthesis may therefore have localized colonization advantages. Removing ROS in the gut abolishes the advantage of these mutants.

**Fig 2:**
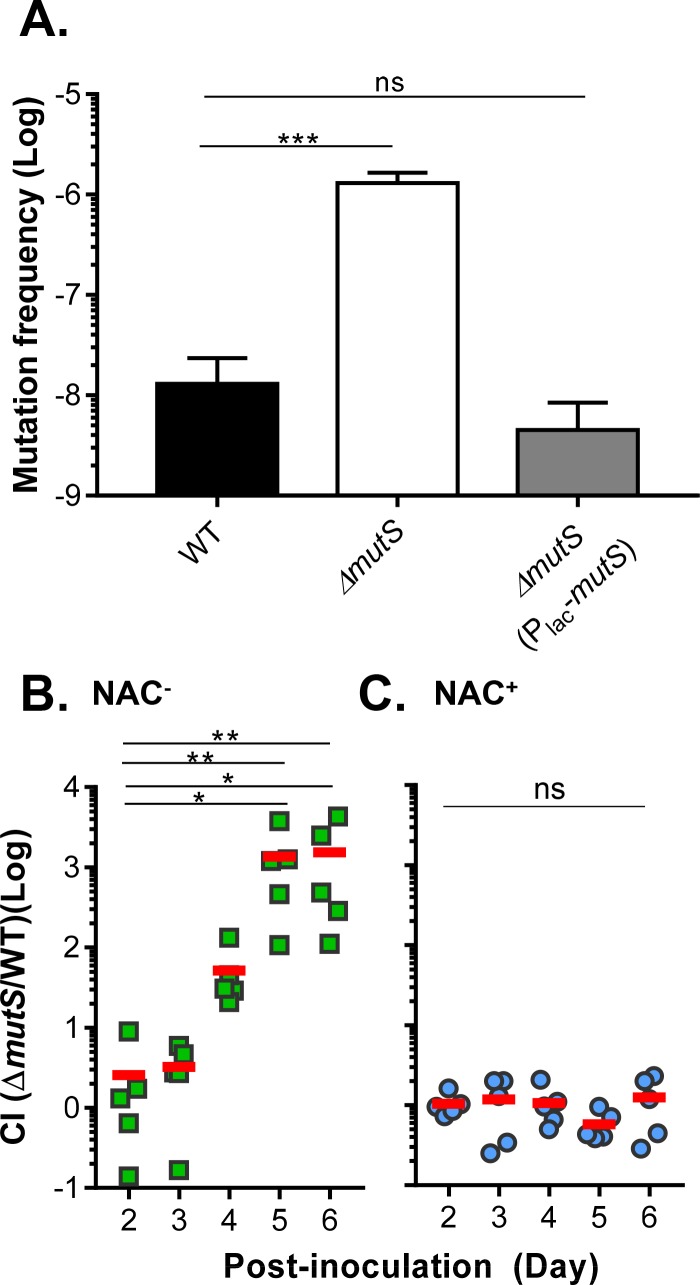
The effect of *mutS* on mutation rate and colonization. **A.** Mutation frequency. Cultures of wildtype, *ΔmutS*, and *ΔmutS* complemented strains were grown in LB until saturation and then plated on LB agar and LB agar + 50 μg/ml rifampicin. After overnight growth at 37°C, rifampicin resistant colonies were scored. Error bars represent means and SDs from three independent assays. ***: One-way ANOVA test, P value < 0.001. ns: no significance. **B&C.** Colonization of in-frame *mutS* deletion mutants. 10^8^ cells of wildtype and *ΔmutS* mutants were mixed in a 1:1 ratio and intragastrically administered to NAC^-^ **(B)** and NAC^+^ **(C)** mice. Fecal pellets were collected from each mouse at the indicated time points and plated onto selective plates. The competitive index (CI) was calculated as the ratio of mutant to wildtype colonies normalized to the input ratio. Horizontal line: mean CI of 5 mice. **: Kruskal-Wallis test, P value < 0.005; *: P <0.05; ns: no significance.

To confirm the Tn-seq results, we constructed an in-frame deletion of *mutS*. We first compared spontaneous rifampicin resistance by colony enumeration of the *ΔmutS* mutant with that of wildtype as a proxy for mutation frequency. As predicted, the mutation frequency in *ΔmutS* mutants was approximately 100-fold higher than that in wildtype (Fig 2A). Complementation of *mutS* on a plasmid restored the *mutS* mutation frequency to wildtype levels (Fig 2A, grey bar). We then performed a competition experiment by mixing differentially-labeled wildtype and *ΔmutS* mutants in a 1:1 ratio and inoculated them into streptomycin-treated mice with NAC treatment as a variable. Fecal pellets were collected daily and colony forming units (CFU) of wildtype and *ΔmutS* mutants were determined by serial dilution and colony enumeration on selective LB agar plates. Fig 2B shows that in the NAC^-^ mice, *ΔmutS* mutants colonized similarly to wildtype initially, but outcompeted wildtype later in the infection. At day 6, the competitive index (*ΔmutS*/WT) exceeded a 1,000-fold advantage. On the other hand, in the NAC^+^ mice, *ΔmutS* mutants did not display a colonization advantage over wildtype throughout the course of infection (Fig 2C). Note that the total number of colonized bacteria was similar between different infection time, mice, and conditions. These data confirm the Tn-seq study suggesting that *ΔmutS* mutants are advantageous over wildtype in NAC^-^ mice, which is predicted to have relatively higher levels of ROS compared to NAC^+^ treated mice.

### Overproduction of catalases in *ΔmutS* mutants isolated from NAC^-^ mice

**Fig 3:**
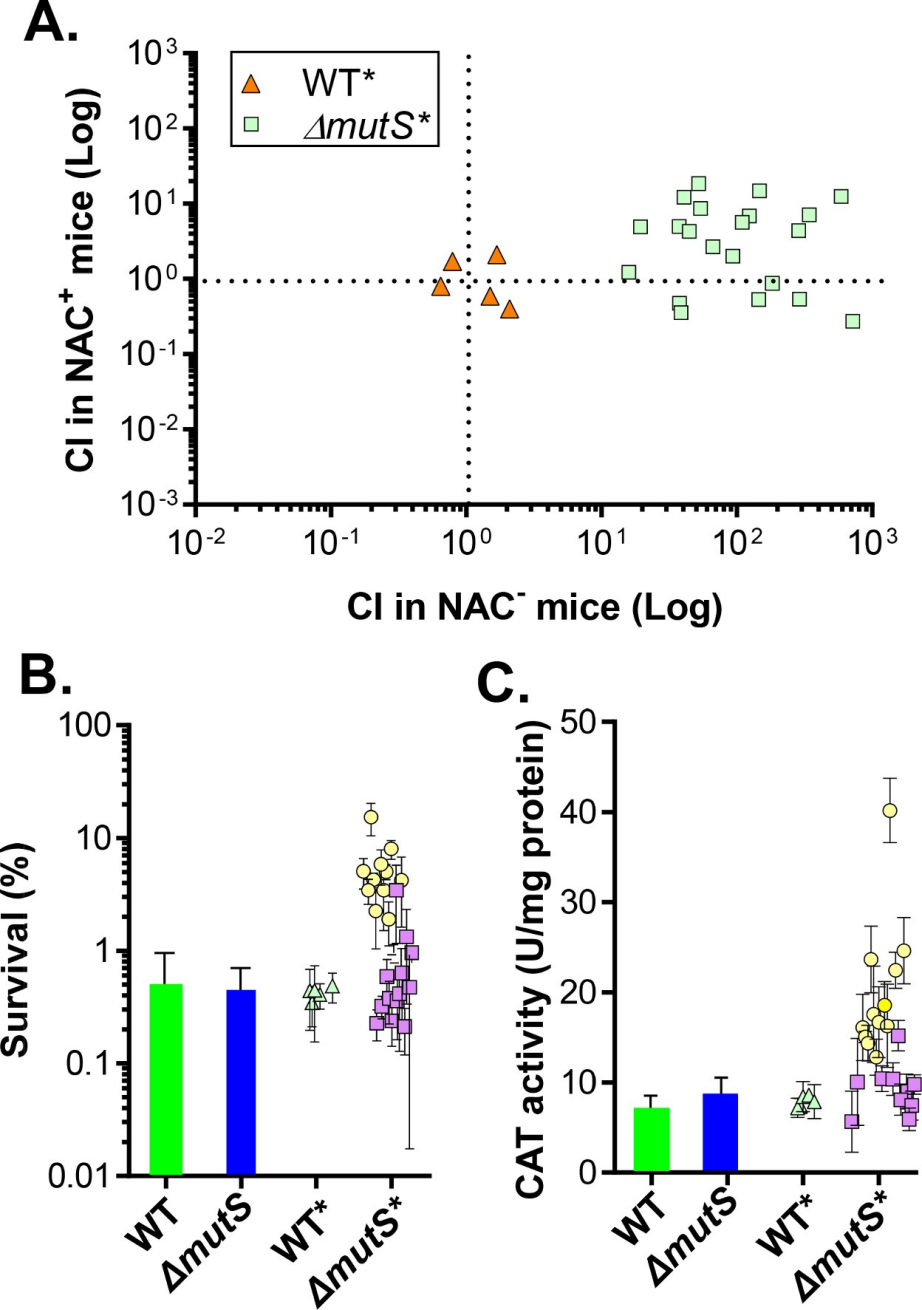
Recolonization and ROS resistance of passaged *V*. *cholerae*. **A.** Competitive index of recolonized isolates. Twenty-four *ΔmutS* mutants isolated from NAC^-^ mice were complemented by a chromosomal copy of *mutS* (ΔmutS*) into the *lacZ* locus. Five wildtype colonies were also selected (WT*) as a control. These isolates were co-infected with wildtype (*lacZ*^*+*^) into 6-week-old CD-1 NAC^-^ and NAC^+^ mice. Fecal pellets were collected after 5 days and plated onto selective plates. The competitive index was calculated as the mutant to wildtype output ratio normalized to the input ratio. One-way ANOVA test P value < 0.001 includes WT* (NAC^+/-^) vs ΔmutS* (NAC^-^) and ΔmutS* (NAC^-^) vs ΔmutS* (NAC^+^). **B.** ROS resistance. Mid-log cultures of wildtype, *ΔmutS*, and *in vivo*-isolated wildtype (WT*), and *ΔmutS* (*lacZ*::*mutS*) (ΔmutS*) were diluted into saline and into saline containing 300 μM H_2_O_2_. After a 1 hr incubation, viable cells were enumerated. Survival rate was calculated by normalizing CFU to the H_2_O_2_-treated group. Error bars represent means and SDs from three independent experiments. **C.** Catalase production. Mid-log cultures were induced with 500 μM H_2_O_2_ for 1 hr. The lysates were then subjected to catalase activity assays. Error bars represent means and SDs from three independent experiments. Circles: smooth variants; squares: rugose colony variants.

To investigate the possible mechanisms that enable a *ΔmutS* colonization advantage in NAC^-^ mice, we further examined these isolates *in vitro* and *in vivo*. To avoid additional accumulation of mutations after *in vivo* passage, we introduced a copy of *mutS* into the *lacZ* locus of *mutS* mutants immediately after being isolated from mice. Introducing the chromosomal copy of *mutS* into *mutS* mutants restored the mutation frequency to wildtype levels (Fig B in S1 Text). We then tested 24 *mutS* (*lacZ*::*mutS*) isolates (annotated as ΔmutS*) from NAC^-^ mice. We first performed competition colonization experiments to examine whether these individual isolates maintain colonization advantages over wildtype. We found that all 24 ΔmutS* tested colonized NAC^-^ mice better than wildtype and the competitive indexes ranged from ~10–1000 (Fig 3A, light green squares). In NAC^+^ mice, these isolates gained little, if any, competitive advantage. As a control, we also tested 5 wildtype isolates (WT*) that were passaged through NAC^-^ mice. These isolates colonized at a comparable level to the wildtype parental strain in both types of mice (Fig 3A, orange triangles). These data suggest that the *ΔmutS* competitive advantage in ROS-rich mice is heritable.

We then measured ROS resistance of these ΔmutS* isolates *in vitro*. Parental *ΔmutS* mutants had a similar *in vitro* growth rate as wildtype in LB medium and AKI virulence-inducing medium [37](Fig C in S1 Text). WT* and ΔmutS* also grew similarly under these conditions (Fig C (C) in S1 Text). When cultured in LB until mid-log and then treated with H_2_O_2_, we found that *ΔmutS* had similar ROS resistance as that of wildtype (Fig 3B). However, approximately half of ΔmutS* isolates tested displayed significantly more resilience to H_2_O_2_ exposure than that of parental *ΔmutS* (Fig 3B circles, one-way ANOVA P value = 0.0005), whereas WT* were similar to the parental wildtype strain (Fig 3B triangles, P value > 0.99). Of note, most of those ΔmutS* isolates that did not produce more catalase displayed different colony morphology (Fig 3B and 3C, squares) (see next section). Correspondingly, about half of ΔmutS* were detected to have more catalase activity (Fig 3C, circles, one-way ANOVA P value = 0.0074). The mutations that led to overproduction of catalase in these *ΔmutS* mutants were not determined. We selected five such high-catalase-producing ΔmutS* isolates and examined transcription of catalase genes (*katG* and *katB*)[14] induced by H_2_O_2_ using qPCR and found transcription of both catalase genes was elevated in three of these mutants (Fig D in S1 Text). For the other two ΔmutS* isolates that did not displayed increasing catalase gene expression, it is possible that mutations involved in post-transcriptional regulation of KatGB activity are accumulated in these isolates. Taken together, these data suggest that mutations leading to increased catalase production are a contributing factor to the observed colonization advantage gained by *ΔmutS* during colonization in NAC^-^ mice. To test this hypothesis, we deleted two catalase genes *katG* and *katB* [14] in *ΔmutS* and the resulting strain was competed with wildtype in NAC^-^ mice. We found that deletion of *katG* and *katB* in *ΔmutS* mutants reduced colonization advantage of *ΔmutS* mutants significantly (Fig E (A) in S1 Text). To further confirm the importance of ROS resistance for *V*. *cholerae in vivo*, we examined the colonization of *ΔoxyR* mutants in NAC^-^ mice. OxyR activates a number of ROS resistance genes in *V*. *cholerae* [14, 16]. Fig E (B) in S1 Text shows that *ΔoxyR* mutants colonized poorly in this mouse model. These results suggest that ROS is important for *V*. *cholerae* colonization of NAC^-^ mice.

### High frequency of arising rugose variants in *ΔmutS* mutants contributes to *in vivo* ROS resistance

**Fig 4:**
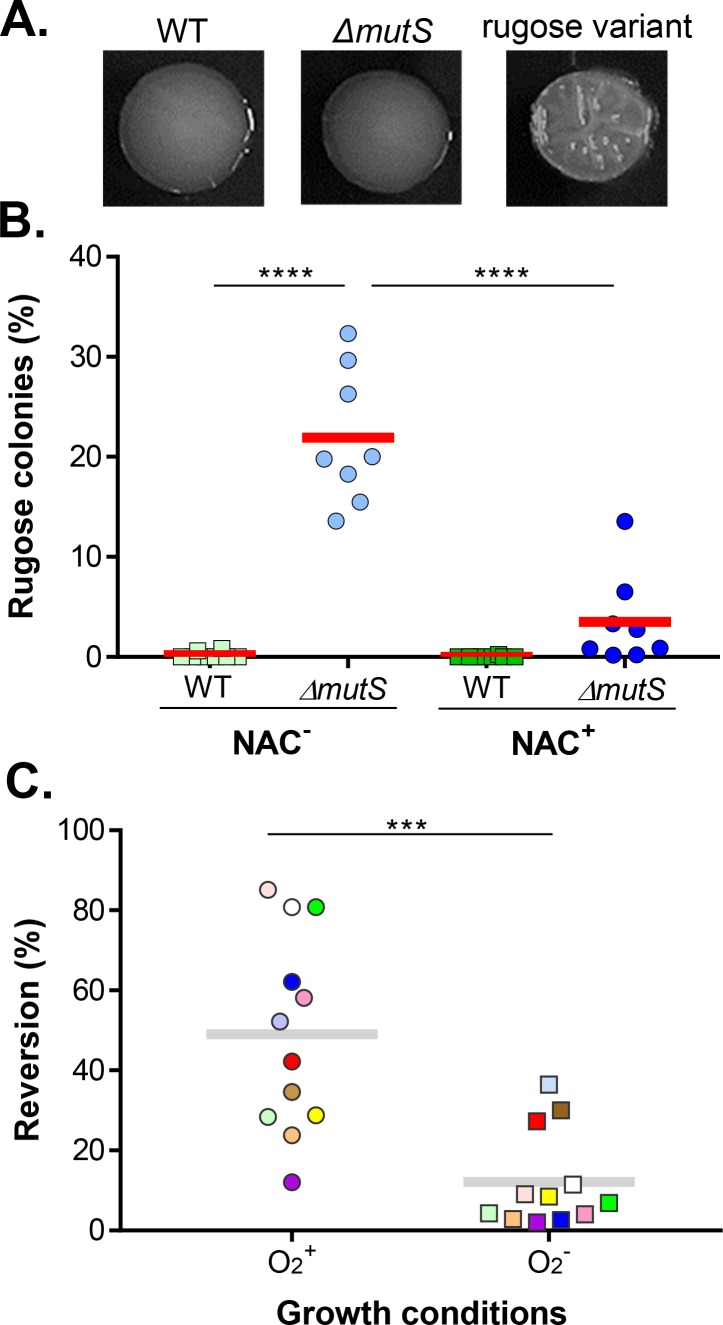
Rugose phenotypes of *V*. *cholerae* isolated from NAC^-^ and NAC^+^ mice. Fecal pellets from 5-day-PI NAC^-^ and NAC^+^ mice were resuspended in PBS and diluted samples were spread onto selective LB plates. After overnight incubation at 37°C, the plates were incubated at room temperature for two days. The colonies were photographed (**A**) and the percentage of rugose colonies was determined (**B**). Each data point represents the percentage of rugose colonies out of at least 300 total colonies isolated from one mouse. Horizontal line: average percentage of 8 mice. ****: One-way ANOVA test, P value <0.0001. **C.** Reversion rate of the ΔmutS* rugose variants. Rugose colonies were resuspended in LB and spread onto selective LB plates. After overnight incubation at 37°C aerobically (circles) and anaerobically (squares), the plates were incubated at room temperature for two days. The percentage of smooth colonies was determined out of at least 400 colonies. Colors correspond to unique isolates. ***: Student t-test P < 0.001.

Upon enumeration of bacteria from fecal mouse pellets, an unusually high number of rugose (wrinkled) colonies, originating from smooth *ΔmutS* mutants, were observed on LB plates (Fig 4A). It has been reported that *V*. *cholerae* can switch its colony morphology from smooth to rugose phenotypes due to the overproduction of exopolysaccharide. This phenotypic switch is reversible and confers greater resistance to environmental stresses compared to strains that undergo this transition at low frequency [38–40]. We thus determined the frequency of rugose colony formation in wildtype and *ΔmutS* isolates from NAC^-^ and NAC^+^ mice. Fig 4B shows that from NAC^-^ mice, a significant number of output *ΔmutS* colonies displayed the rugose phenotype, ranging from ~5% to ~30% of total colonies isolated form each mouse. In NAC^+^ mice, however, the percentage of rugose colonies recovered from *ΔmutS* mutants was much lower (Fig 4B, blue circles). As for wildtype that were isolated from either NAC^-^ or NAC^+^ mice, a relatively low number of colonies displayed the rugose phenotype (Fig 4B, squares). These data suggest that the lack of a functional DNA repair system may increase the frequency of rugose colony formation, which may lead to enhanced survival in ROS-rich, *in vivo* environments. Interestingly, when the rugose variants were cultured in liquid LB with aeration, a majority of them reversed to smooth colonies with high reversion rates (Fig 4C, left panel). However, if incubated anaerobically, which mimics the *in vivo* growth condition, the reversion rates were less prominent as compared to aerobic incubation (Fig 4C, right panel), implying that anaerobiosis may be one of the *in vivo* selective pressures that promote rugose colony formation. These data suggest the involvement of temporal phenotypic switches during *V*. *cholerae* infection possibly mediated or enhanced by genetic adaptation.

**Fig 5:**
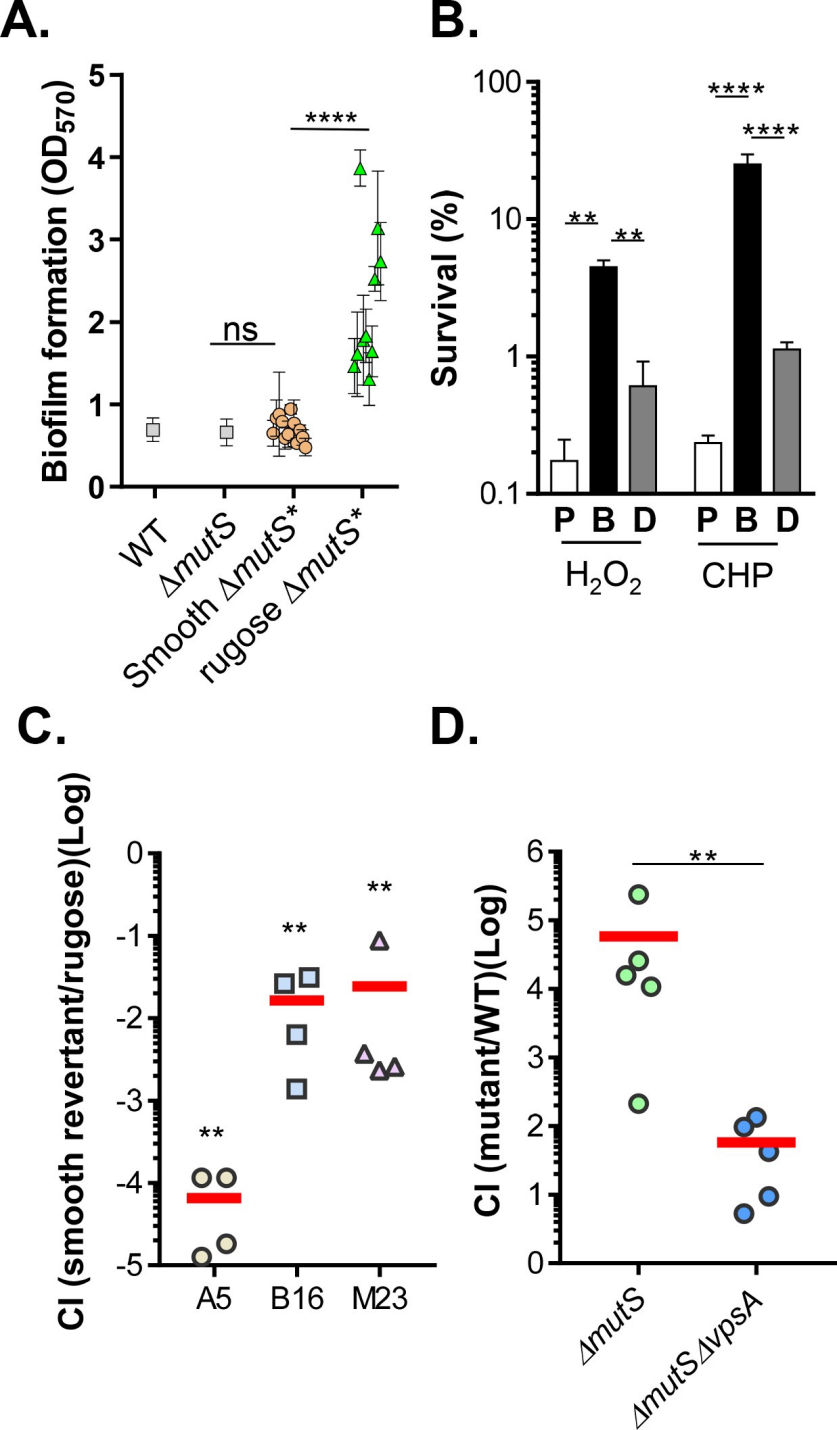
Biofilm formation capacity of passaged isolates and ROS resistance of biofilm-associated cells. **A.** Biofilm formation. Wildtype, *ΔmutS*, and ΔmutS* smooth and rugose variants were cultured in LB without shaking for 16 hrs at 37°C. Culture supernatants were removed, and biofilms were washed with PBS. Biofilm formation was quantified as described previously [9]. Error bars represent means and SDs from three independent experiments. ****: One-way ANOVA test, P value <0.0001. **B.** Biofilm resistance to ROS. Planktonic cells (P), biofilm-associated cells (B), and disrupted biofilm cells (D) were incubated with fresh LB containing 1 mM H_2_O_2_ or 100 μM cumene hydroperoxide (CHP) for 1 hr. The surviving cells were then enumerated by serial dilution and plated onto LB agar. Error bars represent means and SDs from three independent experiments. ****: One-way ANOVA test, P value <0.0001. **: P < 0.05. **C.** Colonization of smooth revertants in NAC^-^ mice. 10^8^ cells of ΔmutS* rugose isolates and their Rif-resistant smooth revertants were mixed in a 1:1 ratio and intragastrically administered to NAC^-^ mice. Fecal pellets were collected from each mouse at 4-day PI and plated onto selective plates. The competitive index (CI) was calculated as the ratio of smooth revertants to parental rugose colonies normalized to the input ratio. Horizontal line: mean CI of 4 mice. **: One-way ANOVA test P < 0.005 [compared with the input ratios (A5 = 1.1±0.7; B16 = 1.7±1.0; M23 = 1.3±0.7)]. **D.** Colonization of biofilm formation-deficiency mutants in NAC^-^ mice. *ΔmutS* or *ΔmutS ΔvpsA* mutants were mixed with wildtype at 1:1 ratio and intragastrically administered to NAC^-^ mice. Fecal pellets were collected from each mouse at 4-day PI and plated onto X-gal plates with appropriate antibiotics. The competitive index (CI) was calculated as the ratio of mutants to wildtype normalized to the input ratio. Horizontal line: mean CI of 4 mice. **: Mann-Whiteney test P value < 0.01.

To determine whether rugose colony phenotypes contribute to enhanced survival, we performed *in vitro* experiments to investigate the possible role of these variants in ROS resistance. We found that a majority of these rugose ΔmutS* variants did not display more ROS resistance in liquid cultures (Fig 3B, squares) and did not display increased catalase production compared to wildtype (Fig 3C, squares). The rugose colony phenotype is often the result of the overproduction of exopolysaccharides, a major component of the biofilm matrix [38, 41]. To examine whether exopolysaccharide overproduction is the cause of rugose colony formation in ΔmutS* isolates, we measured the biofilm formation capacity of various isolates. We found that biofilm mass formed by smooth variants of ΔmutS* was similar to that of wildtype and *ΔmutS* parental strains, whereas rugose variants displayed an increased biofilm formation capacity (Fig 5A). We thus hypothesized that rugose variants are enriched in ROS-rich intestines due to their increased biofilm production and predict that biofilm-associated cells are more resistant to ROS exposure. To test this prediction, we assessed the viability of planktonic and biofilm-associated cells after exposure to organic and inorganic oxidants (Fig 5B). Biofilms were formed on glass test tubes at the air-broth interface through static culture. The majority of planktonic cells were killed after exposure to 1 mM H_2_O_2_ or 100 μM cumene hydroperoxide exposure for 60 mins. In contrast, biofilm-associated cells displayed more than a 30-fold increase in resistance to ROS than planktonic cells (Fig 5B). ROS resistance was mostly eliminated when biofilm structures were disrupted by vortexing with glass beads prior to ROS exposure (Fig 5B, grey bars). These results indicate that it is primarily the physical structure of the biofilm that confers protection against ROS, rather than increased ROS resistance in individual cells. Taken together, our results imply that biofilm formation *in vivo* may play a role in ROS resistance.

Rugose colonies are often caused by mutations in the quorum sensing master regulator HapR and many clinically-isolated rugose variants harbor loss-of-function *hapR* mutations [9, 19]. We examined possible disruptions of the quorum sensing pathway in the rugose ΔmutS* mutants we isolated and found that they were similar to wildtype (Fig F (A) in S1 Text). Sequencing analysis did not reveal any mutations in the *hapR* locus. Indeed, although *ΔhapR* mutants form thicker biofilms [9, 19], *ΔhapR* displayed colonization defects in both NAC^-^ and NAC^+^ mice (Fig F (B) in S1 Text), suggesting that HapR may regulate other targets that are involved in adult mouse colonization. To test our hypothesis that biofilm formation is important for *in vivo* ROS resistance, we first performed *in vivo* competition experiments using ΔmutS* rugose variants and their corresponding smooth revertants. We selected spontaneous rifampicin resistant smooth revertants in order to distinguish them with their parental rugose strains. Fig 5C shows that all three smooth revertants displayed different degrees of colonization disadvantage over their parental rugose ΔmutS* in NAC^-^ mice. The competitive indexes of these rugose/smooth variants were comparable to those indexes when these rugose ΔmutS* isolates competed with wildtype (Fig 3A), suggesting that increasing biofilm formation is the main factor in the rugose isolates that promotes ROS resistance *in vivo*. To further confirm this, we then deleted *vpsA*, which encodes the major component of the Vibrio polysaccharide biosynthesis pathway [42], in *ΔmutS* and the resulting strain was competed with wildtype in NAC^-^ mice. We found that abolishing biofilm formation capacity in *ΔmutS* mutants reduced the colonization advantage of *ΔmutS* mutants significantly (Fig 5D). These data again suggest that biofilm formation *in vivo* may play a role in ROS resistance. Of note, *ΔmutS/ΔvpsA* still outcompeted wildtype. It is possible that accumulation of other beneficial mutations, such as those enhancing catalase production, may elevate ROS resistance *in vivo* for *ΔmutS/ΔvpsA* mutants.

### ROS increases mutation frequency *in vitro* and *in vivo*

**Fig 6:**
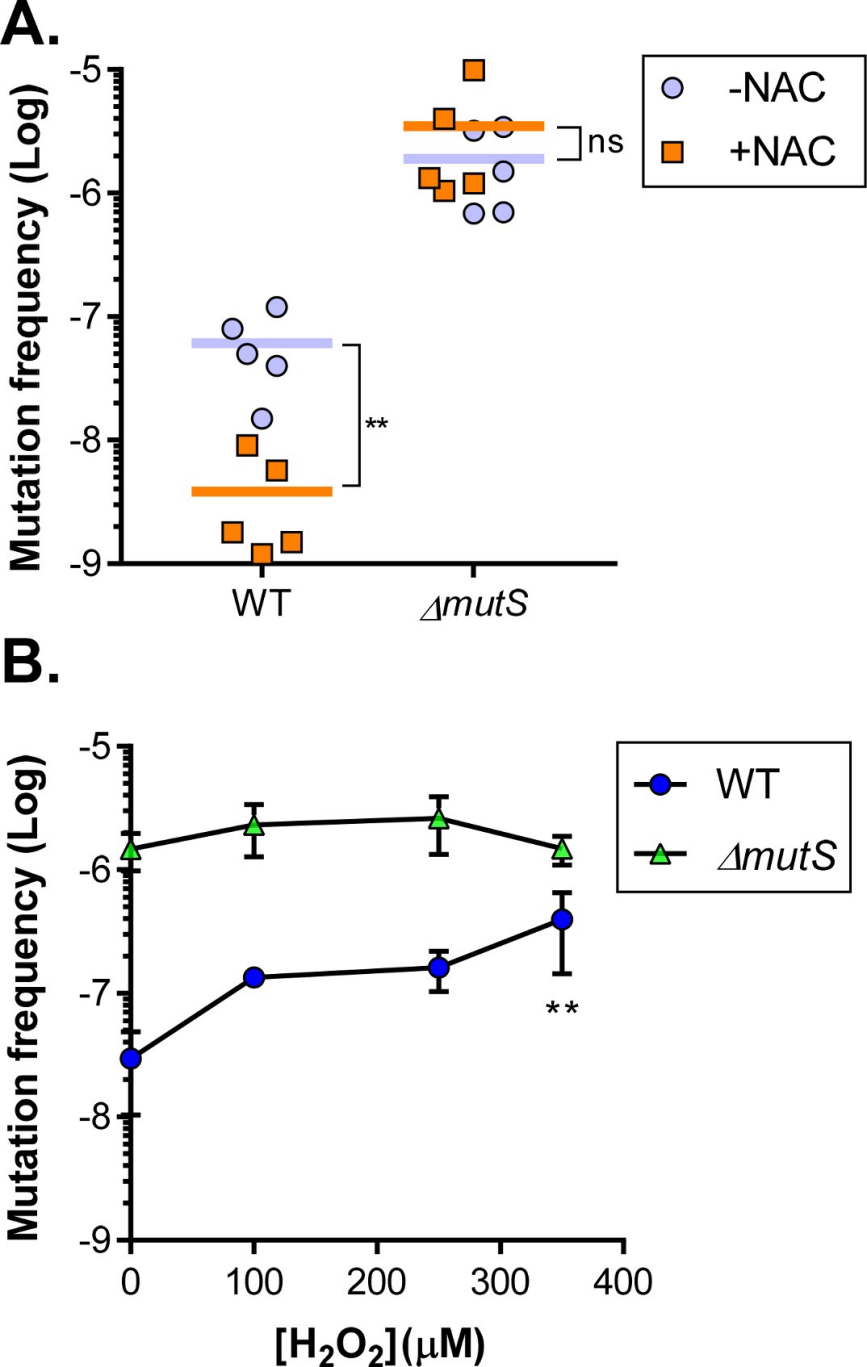
ROS exposure effects on mutation frequency *in vivo* and *in vitro*. **A.** Mutation rate *in vivo*. Fecal pellets from wildtype or *ΔmutS* mutants colonized in NAC^-^ (blue circles) and NAC^+^ (orange squares) mice were collected and homogenized in LB containing streptomycin. After brief centrifugation, the supernatants were incubated at 37°C shaker for 12 hrs. The cultures were then serial diluted onto LB agar + streptomycin and LB agar + rifampicin and streptomycin. After overnight growth at 37°C, rifampicin resistant colonies were scored. **: Mann-Whitney test P < 0.01; ns: no significance. **B.** H_2_O_2_ effects on mutation rate *in vitro*. Overnight cultures of wildtype and *ΔmutS* were inoculated into fresh LB in the presence of indicated concentration of H_2_O_2_ and grown at 37°C shaking for 12 hrs. The cultures were then plated on LB agar and LB agar + 50 μg/ml rifampicin. After overnight growth at 37°C, rifampicin resistant colonies were scored. Error bars represent means and SDs from four independent experiments. **: one-way ANOVA P <0.01 (compared to 0 μM H_2_O_2_).

Mutations in DNA repair systems greatly increase mutation rates in bacteria, as shown by this and other studies, and it has also been reported that ROS enhances mutation frequency in bacteria [43–45]. We then sought to examine whether *V*. *cholerae* may display distinct mutation frequencies as a function of *in vivo* ROS exposure. Both wildtype and *ΔmutS* mutants were inoculated into mice with and without NAC treatment as done in previous experiments. After 3 days of colonization, we collected fecal pellets and outgrew *V*. *cholerae* in LB medium for 12 hrs. We then plated these bacteria on rifampicin to determine mutation rate through a gain of function mutation in *rpoB* that confers resistance to rifampicin. We determined that for wildtype *V*. *cholerae* colonized in NAC^-^ mice, the mutation frequency was over 30-fold higher than those in NAC^+^ mice (Fig 6A). For *ΔmutS* mutants, as expected, the mutation frequency *in vivo* was high, but there was no significant difference between colonizers in NAC^-^ and NAC^+^ mice (Fig 6A), suggesting a theoretical limit of *in vivo* mutagenesis or that the observed elevation in mutation frequency caused by ROS is mediated by a reduction in MMR activity. We also determined the *in vitro* mutation rate in the presence of ROS. Upon exposure to higher levels of H_2_O_2_, elevated mutation frequency was detected in wildtype, whereas changes in mutation rate in *ΔmutS* mutants had no statistical significance (Fig 6B). These data suggest that ROS enhances mutation rate for *V*. *cholerae* in both *in vitro* and *in vivo* environments. This stress-induced mutagenesis and resulting increased genetic variability may provide additional means for *V*. *cholerae* to adapt to ROS-rich environments.

## Discussion

Bacterial pathogens are constantly confronted with changing and aggravating environments and have been known to leverage genetic adaptation as a means to overcome challenges faced in these environments. In this study, we used a streptomycin-treated mouse model to study *V*. *cholerae* ROS resistance *in vivo*. Bacteria experience host-generated oxidative stress in the streptomycin-treated adult mouse model [15, 27, 28]. Inclusion of antioxidant N-acetyl cysteine (NAC) significantly reduced ROS levels [15](Fig G in S1 Text). For mice without the streptomycin treatment, ROS levels were lower than streptomycin-treated mice, but remained detectable (Fig G (A) in S1 Text). In addition, it has been reported that during choleric diarrhea, ROS levels were increased in the host [12, 13]. Taken together, it is suggestive that ROS stress encountered by *V*. *cholerae* in the streptomycin-treated mouse model may be physiologically relevant. By the Tn-seq screen, we discovered that hypermutation rates resulting from the impairment of the *V*. *cholerae* mismatch repair system (*ΔmutS*) led to a colonization advantage in mice, which was not observed in NAC-treated mice. *E*. *coli* colonization studies of mouse intestines have shown that hypermutation is initially beneficial because it allows for a rapid adaptation to the mouse gut environment [26]. However, such strains then experience a loss of fitness due to the constant accumulation of detrimental mutations. To prevent additional detrimental mutations and to be able to study those mutations that conferred a colonization advantage *in vivo*, we complemented *ΔmutS* isolates from NAC^-^ mice immediately after isolation. Further study shows that passage of *ΔmutS* through NAC^-^ mice resulted in the enrichment of catalase-overproducing isolates and a high frequency rugose phenotype. These ΔmutS* isolates remained super-colonizers in NAC^-^ mice but did not gain advantages in NAC^+^ mice (Fig 3A). We also examined infant mouse colonization (Fig H (A) in S1 Text) as well as virulence gene expression (Fig H (B&C) in S1 Text) and found that compared to wildtype, some ΔmutS* isolates were defective in infant mouse colonization and virulence factor production. These results suggest that mutations are specifically selected to overcome ROS stress in the NAC^-^ mice. Indeed, in a previous report [45] by Davies, et al., it was observed that *V*. *cholerae ΔmutS* mutants displayed an approximately 5-fold defect in infant mouse colonization. Considering the short incubation time in infant mouse colonization (18 hrs) and the speculated lack of inflammation in infant mouse gut, it is possible that *ΔmutS* mutants do not experience the same selective pressures as in ROS-rich adult mice. Similarly, in *P*. *aeruginosa*, *ΔmutS* mutants are attenuated in a mouse model of acute infections but are favored in long term persistence of oropharyngeal colonization in cystic fibrosis mice [25].

Many hyper-mutational bacterial pathogens are frequently identified from clinical and environmental isolates, including *E*. *coli*, *Salmonella*, *P*. *aeruginosa*, *Haemophilus influenzae*, *Neisseria meningitidis*, and *Streptococcus pneumoniae* [46]. This is often the case when bacteria need to adapt a new stressful environment. For example, a high percentage of mutators of *P*. *aeruginosa*, *H*. *influenzae*, and *S*. *aureus* were isolated from cystic fibrosis patients who received antibiotic treatments [47]. Infection of a mammalian host is certainly another new environment to adapt to and an increase in genetic variability can help to cope with host defense systems [48]. *V*. *cholerae* hypermutators have also been found in clinical isolates. In a recent study [49], Didelot et al. reported that among 260 *V*. *cholerae* genomes they sequenced and analyzed, 17 isolates have unusually high number of SNPs that are evenly spread throughout their genomes. Further analysis shows that 14 of these 17 genomes possess genetic variations in one or more of four genes in the MMR system and the mutation rate of these strains are significantly increased compared to the others. Interestingly, the majority of these hypermutator strains were isolated between 1961 and 1965, relatively soon after the beginning of the seventh pandemic. The authors cautiously speculated that hypermutators might be causally associated with the rapid spread of the seventh pandemic. In addition, a mobile element is found to insert into the *mutS* gene of a marine Vibrio species, providing a new mechanism for altering the mutation rate [50].

Hypermutation may promote adaptive evolution for bacteria, but the high mutation rate comes at a cost in fitness in the long term [26]. It has been proposed that bacteria may transiently modulate their mutation rates to balance the trade-off between adaption and the accumulation of detrimental mutations [51]. For example, the expression of *mutS* is downregulated by RpoS in response to antibiotic stress, which increases the mutation rate in several bacterial species including *V*. *cholerae* [52]. In *Streptococcus pyogenes*, the integration and excision of a prophage inserted between *mutS* and *mutL* causes a reversible increase in mutation rate in response to the environmental stress [53]. We found that in wildtype *V*. *cholerae*, mutation rate was significantly increased when colonizing NAC^-^ mice compared to NAC^+^ mice (Fig 6A). This finding suggests that *V*. *cholerae* might utilize increased mutation rates as a temporal strategy for adopting advantageous phenotypes during infection of a ROS-rich host. Interestingly, our Tn-seq screen (S1 Data) revealed that *rpoS* mutants failed to colonize NAC^-^ mice but not NAC^+^ mice. Further testing is required to investigate whether the observed increase in mutation rate in NAC^-^ mice is RpoS-mediated. Moreover, the rugose variants isolated from NAC^-^ mice could reverse to smooth colonies *in vitro* (Fig 4C), indicative of use of temporal adaptive strategies by *V*. *cholerae* to combat ROS during infection. Of note, the mechanism of *V*. *cholerae* smooth-rugose phase variation is not clear, but DNA repair pathways have been implicated in phase variation in several species, including *Neisseria gonorrhoeae*, *V*. *parahaemolyticus*, and *Pseudomonas sp*.[54–57]. Interestingly, the high reversion rate from rugose to smooth colonies under aerobic growth (Fig 4C) occurred in *mutS* complemented background (ΔmutS*), suggesting that reversion is not due to DNA mutation. Cell variants in some bacterial species are generated without the burden of mutation, but rather from reverse biostability, which can be controlled by genetic mechanisms such as DNA rearrangement or epigenetic mechanisms such as DNA methylation [58, 59]. Alternatively, the rapid reversion from rugose to smooth *in vitro* even though these cells have been repaired for *mutS* may simply because that the selective pressure for reversion to the smooth variant is remarkably strong during aerobic growth and therefore the reverting mutations arise rapidly. The exact mechanisms of O_2_-dependent rugose-to-smooth phenotypic switch is currently under investigation.

Hypermutable strains are often associated with higher incidences of antibiotic resistance than strains with lower mutations rates. This study proposes a model of *in vivo* temporal hypermutation by mutating MMR and complementing mutants with functional MMR after isolation. This approach allowed for the identification of ROS resistance mechanisms that could be genetically upregulated under ROS stress. It is likely that this approach could be utilized in the context of distinct stressors such as low pH, desiccation, nitrosative stress, etc., revealing likely mechanisms used to overcome those specific environments by comparing mutation spectra or phenotypic changes between experimental groups. In reverse order, MMR mutants could also be used to shed light on stresses experienced in undefined environments by bacteria by associating enriched pathways with stressors. Insight into the mechanisms used to overcome specific stressors could be used to refine antibacterial strategies. This insight would allow for the proactive targeting of arising mutators under treatment, preventing resistant lineages. This application could improve the efficacy of antibacterial agents and reduce the incidence of resistant mutators.

## Materials and methods

### Ethics statement

All animal experiments were carried out in strict accordance with the animal protocols that were approved by the Ethical Committee of Animal Experiments of Nanjing Agricultural University (Permit Number: SYXK (Su) 2017–0007). All efforts were made to minimize animal suffering. Euthanasia was performed by CO2 inhale.

### Strains, plasmids and culture conditions

*V*. *cholerae* El Tor C6706 [60] was used as a parental strain in this study, and was propagated in LB media containing appropriate antibiotics at 37°C, unless otherwise noted. The *mutS* and *dinB* in-frame deletions were constructed by cloning the regions flanking *mutS* or *dinB* into the suicide vector pWM91 containing a *sacB* counter-selectable marker [61]. The resulting plasmids were introduced into *V*. *cholerae* by conjugation and deletion mutants were selected for double homologous recombination events. The construction of *hapR*, *katG*, *katB*, *oxyR*, and *vpsA* mutants has been described previously [14, 19, 62]. The *mutS* overexpression plasmid was constructed by cloning *mutS* coding sequences downstream of the *lac* promoter in pBBR-MCS-3 [63]. Chromosomal complementation of *mutS* was constructed by inserting *mutS* into the *lacZ* locus using pJL1 [64]. AKI medium was used to induce virulence gene expression [37]. Transcriptional *lux* reporters of promoter regions of *tcpA* have been described previously [64]. For growth of *oxyR* mutants on LB plates, 10 μg/ml catalase from bovine liver was included in the medium. When necessary, rugose variants were propagated in LB media without shaking to avoid smooth revertants.

### Mouse colonization

The streptomycin-treated adult mouse model was used to examine *V*. *cholerae* ROS resistance *in vivo* as previously described [15, 27] with the following modifications. Six-week-old CD-1 mice were provided with drinking water or drinking water containing the antioxidant N-acetyl cysteine (NAC) [1% (wt/vol)] for one week. 0.5% (wt/vol) streptomycin and 0.5% aspartame were then added to the drinking water for the remainder of the experiment. Two days after streptomycin treatment, approximately 10^8^ CFU of each of the two differentially-labeled strains (wildtype and mutant) were mixed at a 1:1 ratio and intragastrically administered to each mouse. Fecal pellets were collected from each mouse at the indicated time points, resuspended in LB, serially diluted, and then plated on plates containing 5-bromo-4-chloro-3-indolyl-β-D-galactopyranoside (X-gal) and appropriate antibiotics. The competitive index was calculated as the ratio of mutant to wildtype colonies normalized to the input ratio.

The infant mouse colonization assays were performed as previously described [65] with the following modifications. Briefly, mid-log phase cultures of WT (*lacZ* ^*+*^) and mutants (*lacZ* ^*-*^) were mixed in a 1:1 ratio and approximately 10^5^ cells were intragastrically inoculated into 5-day-old CD-1 suckling mice. After a 20-hr period of incubation, mice were sacrificed. Small intestines were harvested and homogenized, the ratio of mutants to WT bacteria was determined by plating on LB agar containing antibiotics and X-Gal.

### Tn-seq screens to identify *in vivo* ROS resistance-related genes

Approximately 10^8^ CFU from overnight culture of a saturated Tn5 insertion C6706 library using pRL27 [66] were then intragastically inoculated into six-week-old CD-1 mice +/- N-acetyl cysteine (NAC) treatment (5 mice/group). 3 days PI, freshly-collected fecal pellets from each group were pooled and homogenized, the samples were then filtered through a 40 μm membrane. The filtrates were centrifuged, bacterial pellets were resuspended into 20 ml LB medium with appropriate antibiotics and were grown to saturation for DNA extraction (output library). The transposon junctions were amplified from sheared gDNA samples and subjected to massive parallel sequencing using Illumina MiSeq as described previously [6]. All read mapping and data analysis were performed using previously described methods [67].

### ROS resistance and catalase production assays

Overnight cultures of wildtype, *ΔmutS*, and *in vivo*-isolated *mutS* (*lacZ*::*mutS*)(designated *ΔmutS* *) strains were inoculated at 1:100 into fresh LB containing appropriate antibiotics and shaken at 37°C until mid-log phase. Cultures were then diluted into saline and into saline containing 300 μM H_2_O_2_ and were further incubated for 1 hr. Viable cells were then enumerated by serial dilution and plating. Survival rate was calculated by normalizing CFU to the H_2_O_2_-treated group. Catalase production assays used mid-log cultures that were induced with 500 μM H_2_O_2_ for 1 hr. 1 ml of culture samples was withdrawn. Rinsed cells were collected and lysed using sonication. The lysates were then subjected to catalase activity assays using the Fluorometric Catalase Activity Assay Kit (Enco Scientific) per the manufacturer’s instructions. Mid-log cultures were induced with 500 μM H_2_O_2_ for 1 hr for measuring catalase expression. Bacterial cells were then collected and total RNA was extracted using TRIzol (Invitrogen). Single-stranded cDNA was synthesized using SuperScript III reverse transcriptase (Invitrogen) with hexadeoxyribonucleotide mixture as primers. Reverse transcription-quantitative PCR (qRT-PCR) was carried out by using the CFX96 real-time PCR system (Bio-Rad) and a two-step RT-qPCR kit with SYBR green detection (TaKaRa). To standardize results, the relative abundance of 16S rRNA was used as the internal standard.

### Biofilm formation and biofilm ROS resistance assays

Overnight cultures of wildtype, *ΔmutS*, and *in vivo*-isolated ΔmutS* strains were inoculated at 1:100 into fresh LB containing appropriate antibiotics and incubated without shaking at 37°C for 16 hrs. Culture supernatants were removed, and biofilms were washed with PBS. Biofilm formation was quantified by crystal violet staining as previously described [9].

To compare the ROS resistance of planktonic and biofilm associated cells, overnight cultures were inoculated at 1:100 into LB and incubated for 16 hrs at 37°C without shaking. Planktonic cells were removed and pelleted, while the remaining biofilms were rinsed with PBS. Fresh LB containing 1 mM H_2_O_2_ or 100 μM cumene hydroperoxide (CHP) was then added into tubes containing either rinsed biofilms or pelleted planktonic cells and further incubated for 1 hr. To disrupt biofilm structures, cultures were vortexed for 1 minute in the presence of glass beads. The surviving cells were then enumerated by serial dilution and plated onto LB agar.

### Mutation frequency analysis

Overnight cultures of wildtype, *ΔmutS*, and ΔmutS* strains were inoculated into fresh LB containing different concentrations of H_2_O_2_ and grown at 37°C shaking for 12 hrs. The cultures were then plated onto LB agar +/- 50 μg/ml rifampicin. After overnight growth at 37°C, rifampicin resistant colonies were scored. The *in vivo* mutation frequency was determined using the protocol described previously [45] with modifications. Briefly, fecal pellets from *V*. *cholerae* colonized mice were collected and homogenized in 10 ml LB containing 500 μg/ml streptomycin. After brief centrifugation, the supernatants were incubated at 37°C shaking for 12 hrs. The cultures were then serially diluted and plated onto LB agar containing streptomycin (500 μg/ml) and LB agar containing rifampicin (50 μg/ml) and streptomycin (500 μg/ml). After overnight growth at 37°C, rifampicin resistant colonies were scored.

### Virulence gene and quorum sensing regulated gene expression

Overnight cultures of *V*. *cholerae* strains containing P_*tcpA*_-*luxCDABE* transcriptional fusion plasmids were inoculated 1:10,000 into AKI medium [37] and incubated without shaking at 37°C for 4 hrs, followed by shaking at 37°C for an additional 3 hrs. Luminescence was then measured at the indicated time points and normalized to OD_600_. At the final time point, 10^9^ cells were subjected to sodium dodecyl sulfate-polyacrylamide gel electrophoresis (SDS-PAGE) and immunoblotting using anti-TcpA antiserum.

To determine the functionality of HapR-regulated quorum sensing, the cosmid pBB1, carrying the *V*. *harveyi lux* operon [68] was introduced into *V*. *cholerae* strains by conjugation. The resulting strains were grown in LB with appropriate antibiotics at 30°C overnight, diluted to a concentration of 1:100 in fresh LB and transferred to white opaque 96 well plates and incubated at 30°C shaking. Luminescence was read at OD_600_ = 1.

## Supporting information

S1 Data Tn-seq reads of *V. cholerae* colonization in NAC^-^ and NAC^+^ mice. (XLSX)

S1 Text **Fig A**. The effect of *dinB* on colonization and mutation rate. **A&B**. Colonization of in-frame *dinB* deletion mutants. 10^8^ cells of wildtype and *ΔdinB* mutants were mixed in a 1:1 ratio and intragastrically administered to NAC^-^ **(A)** and NAC^+^. **(B)** mice. Fecal pellets were collected from each mouse at the indicated time points and plated onto selective plates. The competitive index (CI) was calculated as the ratio of mutant to wildtype colonies normalized to the input ratio. Horizontal line: mean CI of 4 mice. **B**. Mutation frequency. Cultures of wildtype and *ΔdinB* strains were grown in LB until saturation and then plated onto LB agar and LB agar + 50 μg/ml rifampicin. After overnight growth at 37°C, rifampicin resistant colonies were scored. Error bars represent means and SDs from three independent assays. ns: Student t-test no significance.
**Fig B**. Chromosomal complementation of *mutS*. Cultures of wildtype, *ΔmutS*, and chromosomally inserted (in *lacZ* locus) mutS in *ΔmutS* were grown in LB until saturated and then plated on LB agar and LB agar + 50 μg/ml rifampicin. After overnight growth at 37°C, rifampicin resistant colonies were scored. ****: One-way ANOVA P < 0.0001. ns: no significance.
**Fig C**. The effect of *mutS* on *V. cholerae* growth. Wildtype and *ΔmutS* growth in LB (shaking)(**A**) and AKI medium (standing)(**B**). OD_600_ was measured. **C**. Growth of WT* and ΔmutS* in LB and AKI to mid-log phase. OD_600_ was measured and compared with their parental strains.
**Fig D**. Expression of catalase genes in ΔmutS* isolates. Mid-log cultures of wildtype, *ΔmutS*, and selected *ΔmutS* * were induced with 500 μM H_2_O_2_ for 1 hr. Total RNA was extracted and cDNA was synthesized. Reverse transcription-quantitative PCR (qRT-PCR) was carried out and normalized against 16S rRNA as the internal standard. Error bars represent means and SDs from three independent assays. *: One-way ANOVA P <0.05 (compared to wildtype).
**Fig E**. Colonization of ROS-sensitive mutants in NAC- mice. **A**. *ΔkatGB*. *ΔmutS* or *ΔmutS ΔkatGkatB* mutants were mixed with wildtype at 1:1 ratio and intragastrically administered to NAC- mice. Fecal pellets were collected from each mouse at 4-day PI and plated onto X-gal plates with appropriate antibiotics. The competitive index (CI) was calculated as the ratio of mutants to wildtype normalized to the input ratio. Horizontal line: mean CI of 5 mice. **: Mann-Whiteney test P value < 0.01. B. *ΔoxyR*. *ΔoxyR* mutants were mixed with wildtype in a 1:1 ratio and intragastrically administered to NAC- mice. Fecal pellets were collected from each mouse at 4-day PI and plated onto X-gal plates with 10 μg/ml catalase and appropriate antibiotics. The competitive index (CI) was calculated as the ratio of mutants to wildtype normalized to the input ratio. Horizontal line: mean CI of 5 mice. **: Mann-Whiteney test P value < 0.01.
**Fig F**. The relationship between quorum sensing regulator HapR and rugose variants of ΔmutS*. **A**. pBB1 expression in ΔmutS*. Wildtype, *ΔmutS*, and ΔmutS* rugose variants containing a HapR-regulated *luxCDABE* (pBB1) [68] were grown in LB with appropriate antibiotics at 30°C overnight, diluted to a concentration of 1:100 in fresh LB and transferred to white opaque 96 well plates and incubated while shaking at 30°C. Luminescence was read at OD_600_ = 1. **B**. Colonization. Wildtype and *ΔhapR* were co-inoculated into 6-week-old CD-1 mice with or without NAC treatment. Fecal pellets were collected after 5 days and plated onto selective plates. The competitive index was calculated as the ratio of mutant to wildtype colonies normalized to the input ratio.
**Fig G**. ROS production in adult mouse intestinal tissues. Small intestinal frozen tissue sections from mice with no treatment (**A**, -Sm, -NAC), treated with streptomycin (**B**, +Sm, -NAC), and with streptomycin and N-acetyl cysteine (**C**, +Sm, +NAC) were stained with CM-H_2_DCFDA (Invitrogen) for 60 min at 37°C. Images were taken using a fluorescence microscope (IX81; Olympus). Five randomly selected areas were photographed with the same exposure time. The images were processed using the same fixed threshold in all samples by Slidebook 5.0, and cropped using Adobe Photoshop. Representative images are shown.
**Fig H**. The effects of *mutS* on virulence factor production and infant mouse colonization. **A**. The infant mouse colonization assays. Mid-log phase cultures of WT (*lacZ ^+^*) and mutants (*lacZ ^-^*) were mixed in a 1:1 ratio and approximately 10^5^ cells were intragastrically inoculated into 5-day-old CD-1 suckling mice. After a 20-hr period of incubation, mice were sacrificed. Small intestines were harvested and homogenized, the ratio of mutants to WT bacteria was determined by plating onto LB agar containing antibiotics and X-Gal. **B.&C**. Overnight cultures of wildtype, *ΔmutS* and ΔmutS* containing P_*tcpA*_-*luxCDABE* transcriptional fusion plasmids were inoculated 1:10000 into AKI medium [37] and incubated without shaking at 37°C for 4 hrs, followed by shaking at 37°C for an additional 3 hrs. Luminescence was then measured at the indicated time points and normalized to OD_600_ (**B**). At the final time point, 10^9^ cells were subjected to sodium dodecyl sulfate-polyacrylamide gel electrophoresis (SDS-PAGE) and immunoblotting using anti-TcpA antiserum (**C**).
(PDF)
