# Supplementary material for: Hypermutation-induced in vivo oxidative stress resistance enhances Vibrio cholerae host adaptation
Source: PLoS Pathog. 2018 Oct 30;14(10):e1007413. doi: 10.1371/journal.ppat.1007413 (PMC6226196; doi:10.1371/journal.ppat.1007413)
Supplement: S1 Text — Fig A. The effect of dinB on colonization and mutation rate. A&B. Colonization of in-frame dinB deletion mutants. 108 cells of wildtype and ΔdinB mutants were mixed in a 1:1 ratio and intragastrically administered to NAC- (A) and NAC+. (B) mice. Fecal pellets were collected from each mouse at the indicated time points and plated onto selective plates. The competitive index (CI) was calculated as the ratio of mutant to wildtype colonies normalized to the input ratio. Horizontal line: mean CI of 4 mice. B. Mutation frequency. Cultures of wildtype and ΔdinB strains were grown in LB until saturation and then plated onto LB agar and LB agar + 50 μg/ml rifampicin. After overnight growth at 37°C, rifampicin resistant colonies were scored. Error bars represent means and SDs from three independent assays. ns: Student t-test no significance. Fig B. Chromosomal complementation of mutS. Cultures of wildtype, ΔmutS, and chromosomally inserted (in lacZ locus) mutS in ΔmutS were grown in LB until saturated and then plated on LB agar and LB agar + 50 μg/ml rifampicin. After overnight growth at 37°C, rifampicin resistant colonies were scored. ****: One-way ANOVA P < 0.0001. ns: no significance. Fig C. The effect of mutS on V. cholerae growth. Wildtype and ΔmutS growth in LB (shaking)(A) and AKI medium (standing)(B). OD600 was measured. C. Growth of WT* and ΔmutS* in LB and AKI to mid-log phase. OD600 was measured and compared with their parental strains. Fig D. Expression of catalase genes in ΔmutS* isolates. Mid-log cultures of wildtype, ΔmutS, and selected ΔmutS * were induced with 500 μM H2O2 for 1 hr. Total RNA was extracted and cDNA was synthesized. Reverse transcription-quantitative PCR (qRT-PCR) was carried out and normalized against 16S rRNA as the internal standard. Error bars represent means and SDs from three independent assays. *: One-way ANOVA P <0.05 (compared to wildtype). Fig E. Colonization of ROS-sensitive mutants in NAC- mice. A. ΔkatGB. ΔmutS or ΔmutS ΔkatGkatB m [file ppat.1007413.s002.pdf]

Supplemental Figures

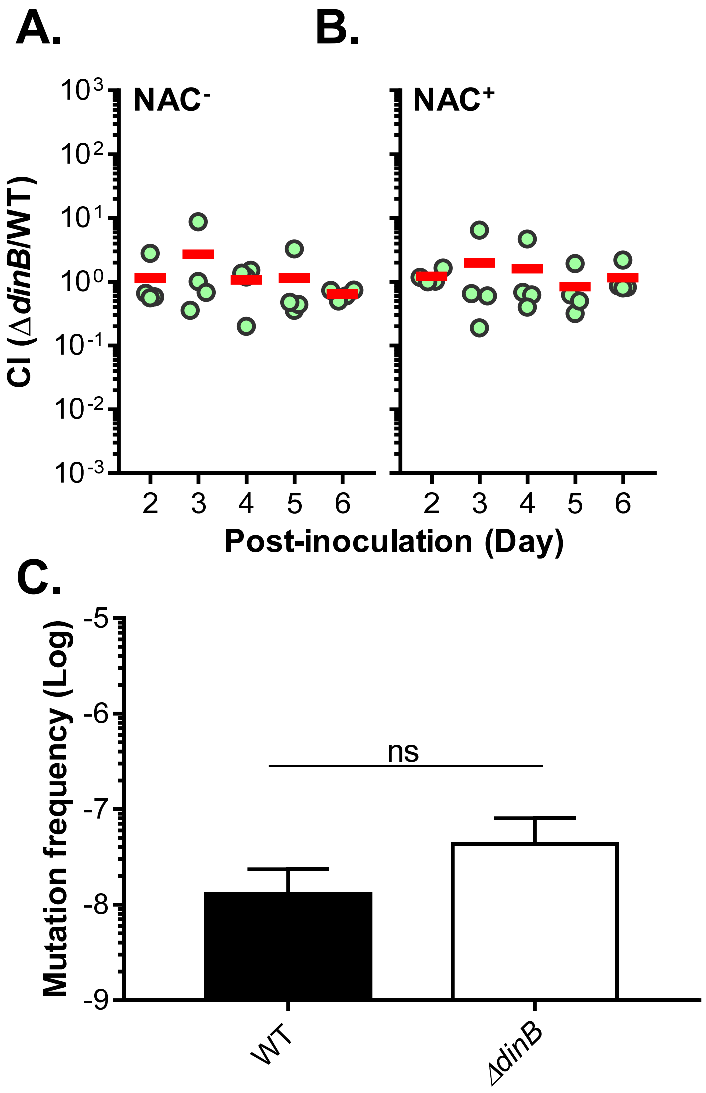

Fig. A. The effect of *dinB* on colonization and mutation rate. **A&B.** Colonization of in-frame *dinB* deletion mutants. 10^8 cells of wildtype and Δ*dinB* mutants were mixed in a 1:1 ratio and intragastrically administered to NAC^- **(A)** and NAC^+. **(B)** mice. Fecal pellets were collected from each mouse at the indicated time points and plated onto selective plates. The competitive index (CI) was calculated as the ratio of mutant to wildtype colonies normalized to the input ratio. Horizontal line: mean CI of 4 mice. **B.** Mutation frequency. Cultures of wildtype and Δ*dinB* strains were grown in LB until saturation and then plated onto LB agar and LB agar + 50 µg/ml rifampicin. After overnight growth at 37˚C, rifampicin resistant colonies were scored. Error bars represent means and SDs from three independent assays. ns: Student t-test no significance.

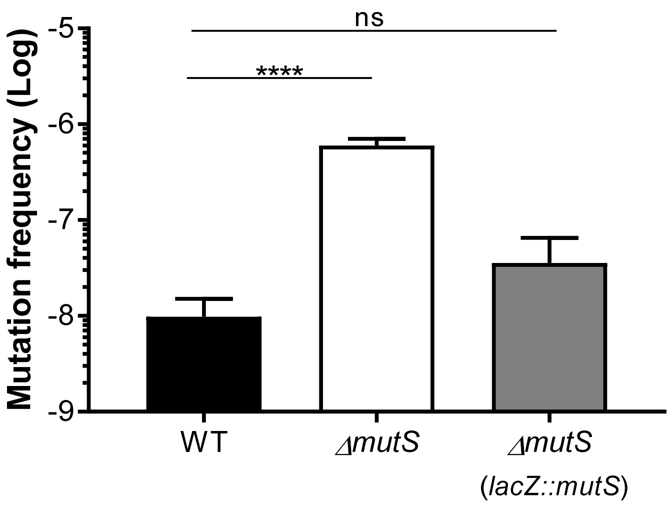

Fig. B. Chromosomal complementation of *mutS*. Cultures of wildtype, Δ*mutS*, and chromosomally inserted (in *lacZ* locus) *mutS* in Δ*mutS* were grown in LB until saturated and then plated on LB agar and LB agar + 50 µg/ml rifampicin. After overnight growth at 37˚C, rifampicin resistant colonies were scored. ****: One-way ANOVA $P < 0.0001$. ns: no significance.

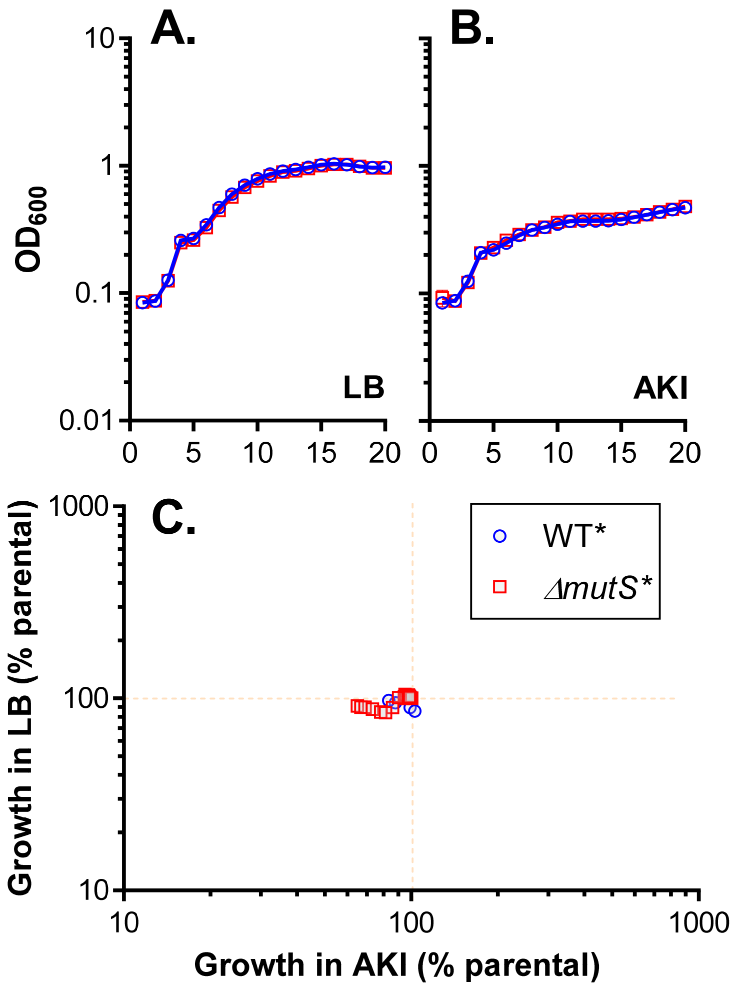

Fig. C. The effect of *mutS* on *V. cholerae* growth. Wildtype and Δ*mutS* growth in LB (shaking)(**A**) and AKI medium (standing)(**B**). OD_{600} was measured. **C.** Growth of WT* and Δ*mutS** in LB and AKI to mid-log phase. OD_{600} was measured and compared with their parental strains.

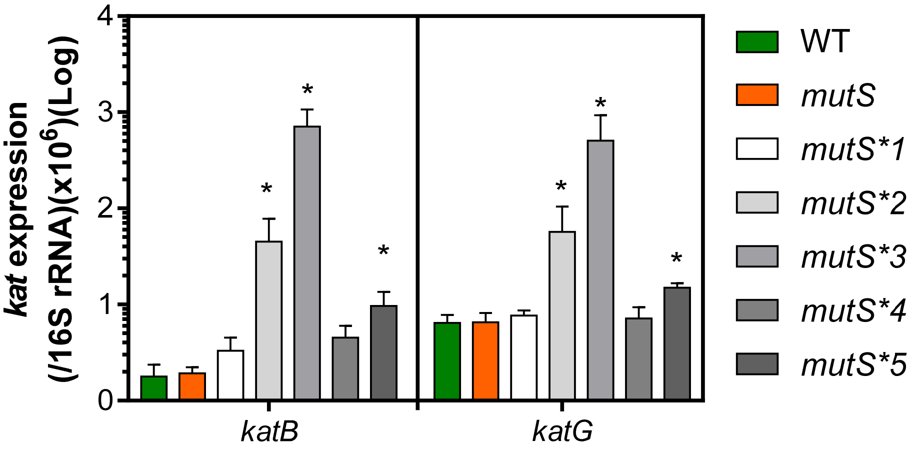

Fig. D. Expression of catalase genes in *ΔmutS** isolates. Mid-log cultures of wildtype, *ΔmutS*, and selected *ΔmutS* * were induced with 500 µM H_2O_2 for 1 hr. Total RNA was extracted and cDNA was synthesized. Reverse transcription-quantitative PCR (qRT-PCR) was carried out and normalized against 16S rRNA as the internal standard. Error bars represent means and SDs from three independent assays. *: One-way ANOVA $P < 0.05$ (compared to wildtype).

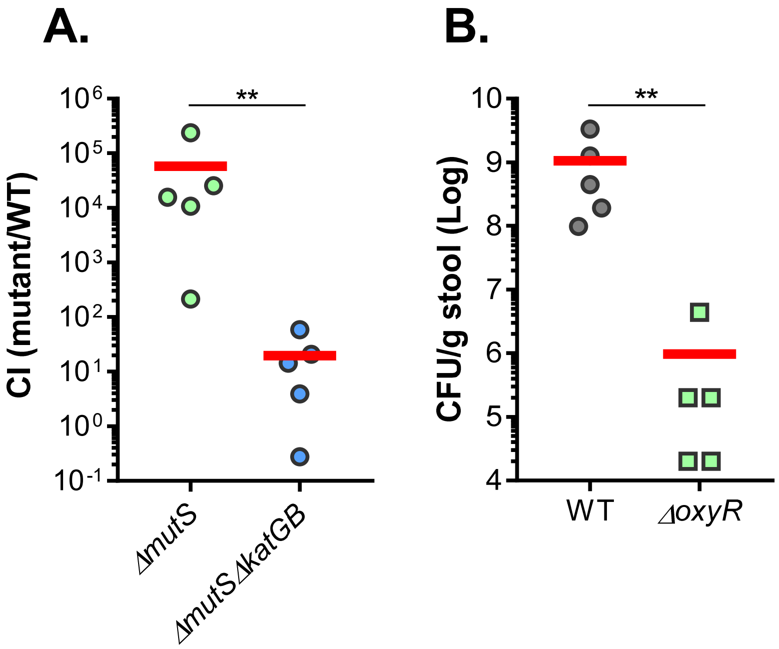

Fig. E. Colonization of ROS-sensitive mutants in NAC- mice. **A.** *ΔkatGB*. Δ*mutS* or Δ*mutS* Δ*katGkatB* mutants were mixed with wildtype at 1:1 ratio and intragastrically administered to NAC- mice. Fecal pellets were collected from each mouse at 4-day PI and plated onto X-gal plates with appropriate antibiotics. The competitive index (CI) was calculated as the ratio of mutants to wildtype normalized to the input ratio. Horizontal line: mean CI of 5 mice. **: Mann-Whiteney test P value < 0.01. B. *ΔoxyR*. Δ*oxyR* mutants were mixed with wildtype in a 1:1 ratio and intragastrically administered to NAC- mice. Fecal pellets were collected from each mouse at 4-day PI and plated onto X-gal plates with 10 µg/ml catalase and appropriate antibiotics. The competitive index (CI) was calculated as the ratio of mutants to wildtype normalized to the input ratio. Horizontal line: mean CI of 5 mice. **: Mann-Whiteney test P value < 0.01.

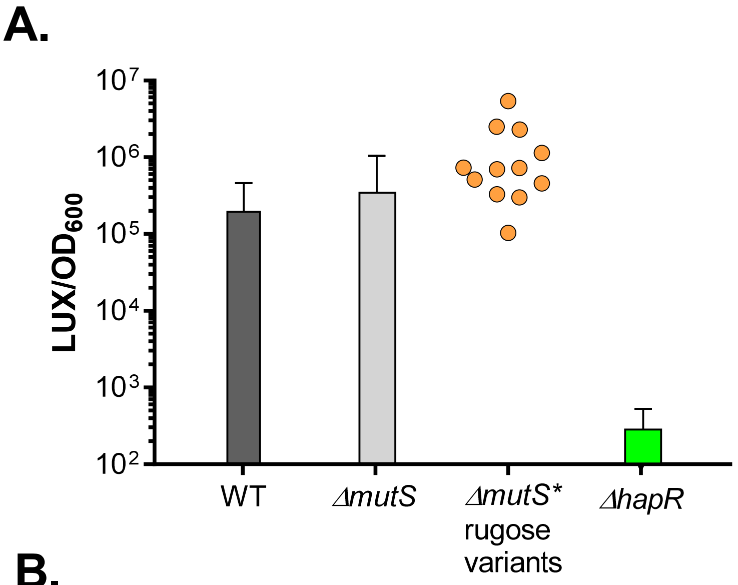

Fig. F. The relationship between quorum sensing regulator HapR and rugose variants of Δ*mutS**. **A.** pBB1 expression in Δ*mutS**. Wildtype, Δ*mutS,* and Δ*mutS** rugose variants containing a HapR-regulated *luxCDABE* (pBB1) [68] were grown in LB with appropriate antibiotics at 30ºC overnight, diluted to a concentration of 1:100 in fresh LB and transferred to white opaque 96 well plates and incubated while shaking at 30ºC. Luminescence was read at OD_{600} = 1. **B.** Colonization. Wildtype and Δ*hapR* were co-inoculated into 6-week-old CD-1 mice with or without NAC treatment. Fecal pellets were collected after 5 days and plated onto selective plates. The competitive index was calculated as the ratio of mutant to wildtype colonies normalized to the input ratio.

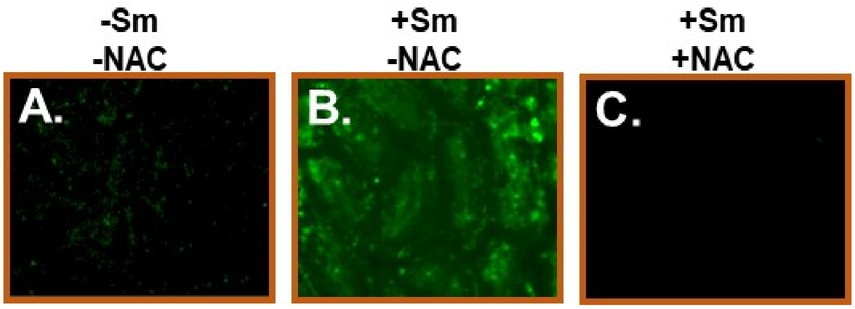

Fig. G. ROS production in adult mouse intestinal tissues. Small intestinal frozen tissue sections from mice with no treatment (**A**, -Sm, -NAC), treated with streptomycin (**B,** +Sm, -NAC), and with streptomycin and N-acetyl cysteine (**C,** +Sm, +NAC) were stained with CM-H_2DCFDA (Invitrogen) for 60 min at 37 ˚C. Images were taken using a fluorescence microscope (IX81; Olympus). Five randomly selected areas were photographed with the same exposure time. The images were processed using the same fixed threshold in all samples by Slidebook 5.0, and cropped using Adobe Photoshop. Representative images are shown.

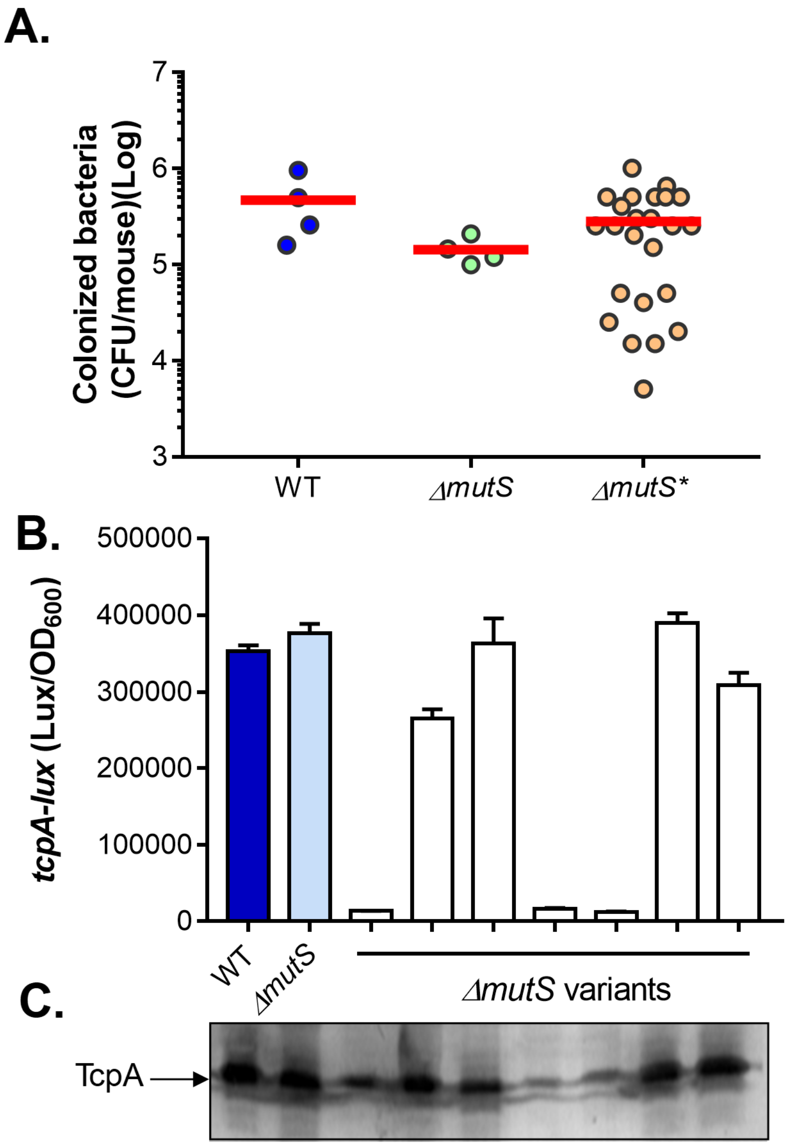

Fig. H. The effects of *mutS* on virulence factor production and infant mouse colonization. **A.** The infant mouse colonization assays. Mid-log phase cultures of WT (*lacZ* $^{+}$) and mutants (*lacZ* $^{-}$) were mixed in a 1:1 ratio and approximately 10^5 cells were intragastrically inoculated into 5-day-old CD-1 suckling mice. After a 20-hr period of incubation, mice were sacrificed. Small intestines were harvested and homogenized, the ratio of mutants to WT bacteria was determined by plating onto LB agar containing antibiotics and X-Gal. **B.&C**. Overnight cultures of wildtype, Δ*mutS* and Δ*mutS** containing P_{tcpA}-*luxCDABE* transcriptional fusion plasmids were inoculated 1:10000 into AKI medium [37] and incubated without shaking at 37°C for 4 hrs, followed by shaking at 37°C for an additional 3 hrs. Luminescence was then measured at the indicated time points and normalized to OD_{600} (**B**). At the final time point, 10^9 cells were subjected to sodium dodecyl sulfate-polyacrylamide gel electrophoresis (SDS-PAGE) and immunoblotting using anti-TcpA antiserum (**C**).
